# Features and spatial effects of urban development and decline in resource-oriented cities: The case of Jilin, China

**DOI:** 10.1371/journal.pone.0289804

**Published:** 2023-08-16

**Authors:** Xincheng Zhu

**Affiliations:** Kelly Hill School, Shanghai, China; Xiangtan University, CHINA

## Abstract

Transforming resource-based cities into sustainable economic development is a great challenge for policy-makers in many countries. However, the economic-centered evaluation system tends to breed the undesirable view of "GDP only" or “brown growth” in the previous case studies which is inconsistent with the long-run and sustainable development of resource-based cities. To fill in this research gap, this paper takes Jilin province in northeast China as a case study to explore urban development problems faced by major resource-based cities during resource depletion. This research constructs a stratified indicator system and conducts an in-depth analysis of the features and spatial effects of urban decline. For this analysis, this paper jointly uses the methods of entropy-weighted TOPSIS, analytic hierarchical process (AHP), and spatial effect model based on a database from 2000 to 2019. The findings of this study show that the current transformation of resource-based cities in Jilin province is generally ineffective and difficult to maintain long-run and sustainable development due to its historical reasons and industrial development background. According to the results, the resource-based cities in Jilin province show an unstable development because of factors such as barriers to the physical renewal of resources, rigid industrial structure, insufficient backup resources, and institutional and policy constraints. Also, the transformation of these cities into sustainable economic development is still facing demographic, social, and ecological difficulties.

## Introduction

A resource-based city is a type of city where the leading industry is extracting and processing natural resources such as minerals and forests [1]. This kind of city is a regional economic center with general urban attributes. These cities show specific characteristics and levels of sustainable development including economic, social, and ecological pillars due to their strong dependence on resources [2–4], showing a unique pattern of cyclical development. Sustainable Development Goal 11 (SDG 11) of the United Nations (UN) is “Sustainable cities and communities” which highlights the discussion on resource-based cities [5]. In general, these cities have three categories including early-stage cities, middle-stage cities, and resource-depleted cities. These cities often experience the process of "construction—prosperity—decline—transformation and revitalization or extinction”.

The industrial transformation of resource-based cities is an unsolved global problem. Previous studies focused on psychology, sociology, economics, and urban development cycles of resource-based cities. For example, Marsh studied the sense of community belonging among residents of coal towns in Pennsylvania, USA, and found that with the mass exodus of people from declining mining areas, about one-third of the people still chose to remain and had a strong sense of community belonging [6]. Also, Bradbury studied the demographic characteristics of resource-based towns in Labrador, Quebec, Canada, and revealed that the economic cycles of extractive industries have profoundly impacted demographic changes in the mining towns [7]. The primary division of resource-based cities’ development patterns is based on mineral processing and utilization [8] as well as labor population and ethnic status [9]. Researchers have put forward the theory of mining areas’ four, five, or six-stage cycle [10]. After the mid-1980s, research on resource-based towns began to diversify, focusing on corporate relationships, labor market structures, and urban transformation. Hayter and Barnes discovered that resource-based industries in Canada have undergone two stages of labor market segmentation [11], and Bradbury proposed transitional responses such as establishing early warning systems, developing financial assistance, and job transition training [12].

In China, researchers have shown that the resource-based cities in the Northeast significantly contributed to economic development. From the founding of New China to the present, resource-based cities have produced 52.9 billion tons of raw coal, 5.5 billion tons of crude oil, 5.8 billion tons of iron ore, and 2 billion cubic meters of timber. During the "First Five-Year Plan" period, 53 out of 156 national key construction projects were laid out in resource-based cities, accounting for nearly half of the total investment, which made historic contributions to the establishment of China’s independent and complete industrial system and economic development.

However, resource-based economies have long suffered from the single industry and unbalanced structure [13–15]. After long-term high-intensity mining, resource reserves continue to decrease, making the cities’ economic development seriously hampered, and industrial transformation and upgrading are imminent. The "National Sustainable Development Plan for Resource-oriented Cities (2013–2020) " published in 2013 by the General Office of the State Council defines national resource-based cities as cities with the development and processing of minerals, forests, and other resources in the region as their leading industries. This plan describes 262 "resource-based cities" for the first time and puts forward the overall objectives and specific requirements for promoting sustainable development in resource-based cities.

Many researchers have focused on the definition and classification of resource-based cities [16, 17], such as the analysis of development dilemmas [18, 19] and the choice of urban transformation paths [20], specifically in China. Since 2010, as resource-based cities have achieved specific results in their transformation development, research has begun to shift to urban spatial patterns and planning responses [21], industrial development [22], and urban competitiveness [23]. However, few studies concentrated on evaluating urban transformational development and exploring problems in its development process in China.

In terms of the research scope, these studies mostly consider the national level or a single typical resource-depleted city as the research object, disregarding the northeastern region with broad coverage, complete types, and typical problems, especially in the resource-based cities in Jilin province. Jilin is one of the provinces located in Northeast China, also known as the Northeast Region. The resource-based cities in the Northeast have played a critical role in China’s economic growth since the founding of New China. These cities are rich in natural resources such as coal, oil, iron ore, and timber, which have been instrumental in the establishment of China’s independent and complete industrial system.

The selection of Jilin Province as the research subject is primarily due to its unique characteristics of development. In comparison with mining towns in the USA and the Quebec region in Canada, China’s resource-based cities have already experienced economic decline before China became a developed country. This provides an excellent sample for studying the transformation issues of resource-based cities in emerging economies.

To fill this research gap, this paper evaluates the development of resource-based cities in Jilin province from 2000 to 2019 by constructing a modern development assessment system. In addition, this paper analyzes the effects, problems, and causes of development in these cities. In this way, this study provides a theoretical basis and practical support for the realization of comprehensive transformation and development of similar resource-based cities not only in Jilin province but also in China and the world.

The primary indicators used in the study were population, industry, society, investment, and ecology. These factors were used as primary indicators in the study to evaluate the development of resource-based cities in Jilin province. The study found that these factors are interrelated and contribute to the unstable development of resource-based cities. For example, barriers to the physical renewal of resources, rigid industrial structure, insufficient backup resources, and institutional and policy constraints were identified as factors that hinder the transformation of resource-based cities into sustainable economic development. Ecological difficulties were also identified as a challenge that resource-based cities face in this transformation. For example, Jilin province is home to several major rivers, including the Songhua River, which is an important source of water for both industrial and residential use. Water pollution can lead to the degradation of these resources, impacting both the local environment and wildlife. This can make the area less attractive for tourism and recreation, further contributing to economic decline.

The results of the paper show that the current transformation of resource-based cities in Jilin province is generally ineffective and difficult to maintain long-run and sustainable development due to historical reasons and industrial development background. The resource-based cities in Jilin province show an unstable development because of factors such as barriers to the physical renewal of resources, rigid industrial structure, insufficient backup resources, and institutional and policy constraints. The transformation of these cities into sustainable economic development is still facing demographic, social, and ecological difficulties.

## Literature review

### Development of resource-based cities

Based on the urban development cycle, resource-based cities have six development stages: construction, employment, transition, maturity, decline, and complete abandonment [24]. This classification provides theoretical references for the formulation of policies on resource-based cities in different periods.

Based on global economic integration, researchers have laid a solid research foundation for developing resource-based cities from the perspectives of human resources, natural resources, capital flows, space, and industrial upgrading. For example, Luis Suazervilla [25] and geologist Hubert divided the life cycle of the resource cities into four phases: 1) preparatory phase, i.e., the stage of preparation before resource development; 2) growth phase, i.e., the stage of a full-scale production to design scale; 3) maturity phase, i.e., after production reaches the scale stage, it continues to develop related industries by using the forward, backward, and side-linkages of the leading industry; 4) Transformation period, that is, the status of mining-oriented industries gradually decline. If new industries emerge at this time, the mineral city will evolve into a comprehensive city; otherwise, it will face decline [26]. In China, resource-based cities emerged mostly from the 1950s to the early 1980s, peaked from the mid-1980s to the mid-1990s, and began to decline in the late 1990s. These studies show that resource-based cities should divert to using the accumulated capital, technology, and talents in the preparatory or growth stage. In this way, these cities can gradually shift their focus to cultivating non-resource-based pillar industries and reduce their dependence on resources to smoothly transform into the mature stage. Cities that have missed the time of transformation should focus on solving the contradictions in various pillars of sustainable development involving economy, society, and environment. In addition, these cities should actively cultivate alternative industries with policy support to expand the development field and realize the transformation.

### Research on the development of resource-oriented cities

Research on the development and transformation of resource-based cities has concentrated on case studies and evaluating the effects of transformation development. For example, Yang Shuying et al. studied the comprehensive remediation of the mine subsidence area in Heze, Shandong Province of China [27]. Also, Lu Wanhe et al. discussed the portability of the "Liaoyuan model" and the ways of transplantation [28]. Chen Guisheng empirically reviewed the role of government in the transformation process of the Ruhr region [29]. Many studies provide a comparative analysis of the transformation of two different resource-based regions. For instance, He Xiong et al. conducted a comparative study on the transformation models of Songyi in Hubei and Fuxin in Liaoning [30]. Moreover, Xue Yi summarized the successful transformation experiences of foreign oil resource cities such as Houston, Calgary, and Sylvia. Y. He conducted a comparative study on the economic restructuring of the mining resource cities of Daqing and Pingxiang based on the theory of urban contraction and resource-based economy [31, 32].

Furthermore, previous studies explored the evaluation of transition effects on various aspects of economic and social transformation, environmental efficiency, and resource and energy use efficiency. Papyrakis et al. argued that the adverse and indirect effects of natural resources on economic growth outweigh the positive and direct effects [33]. Tonts et al. argued that resource dependence tends to shift cities to single resource use, and diversification is relatively tricky [34]. Tan Xuhong et al. analyzed the factors influencing the transformation and development of resource-based cities in the Heilongjiang province of China, and evaluated their green transformation efficiency, suggesting improvements in the industrial system, technological innovation, and ecological civilization [35]. Chang Yu et al. [36] analyzed the challenges faced by the resource-depleted city of Yichun in China in the transformation process based on a review of China’s resource-based city transformation policies and applied an eco-efficiency approach to Huiming. In addition, Zhang et al. analyzed the transformation efficiency of 37 resource-depleted cities in China from 2004 to 2014 using panel data [37]. Chen Yan et al. cited indicators such as environmental regulation, resource dependence, and ownership structure to compare the spatial and temporal evolution and influencing factors between resource-based and non-resource-based cities in Northeast China in terms of the rationalization and advanced levels of the industrial structure by multiple regression and other methods [38]. Also, Fang Xingcun et al. measured the economic transformation efficiency of 23 resource-declining cities in China using [39]. Their studies used a data envelopment analysis (DEA) model and concluded that the transformation efficiency of resource cities in China is not high at present. Moreover, Sun Wei et al. analyzed the transformation efficiency of resource cities in China using the DEA and Malmquist productivity index and found that fewer cities reached the optimal overall efficiency, and scale efficiency was the most important influencing factor of transformation [40].

There have been also various studies on the transformation of resource-based cities. Yu, X. (2020) Proposed an evaluation system for sustainable urban spatial development in regions with underdeveloped economies but rich in ecological resources. The system is based on a systematic coupling analysis of collaborative evaluation information and a quantitative analysis of the influences of urban space elements on sustainable urban development. It was found that among the 14 indicators investigated, industrial land had the greatest impact on sustainable urban spatial development. Other factors such as green area, urban built-up land area, forestry area, and others were found to have a secondary effect on sustainable urban spatial development. B. Marsh and O’faircheallaigh C [6, 41] studied a series of problems encountered in the transformation process of resource-based cities from sociological and demographic perspectives, respectively. Lucas’s four-stage theory of the development of a single industrial community [24] and the later modified and refined six-stage theory of the development of resource-based areas are regarded as the "originators" of the life cycle theory of resource-based towns. Houghton’s long-distance commuting model provides lessons on how resource-based cities can avoid the decline and transformation of mining areas [42]. Bradbury used capital accumulation theory, capital internationalization theory, and uneven development theory to explain the problems faced by resource-based communities for understanding the root causes of these issues. This Canadian scholar later discussed the trajectory of the boom and decline of resource-based industries in the global context and the general pattern of resource-based industry restructuring through an empirical analysis of the coal mining region in western Canada., Bradbury gave policy recommendations such as establishing an early warning system for urban resource depletion and various insurance mechanisms [43].

### Research gap

As the transformation of resource-based cities continues to progress, researchers have increasingly concentrated on case studies and comparative analysis of resource-based cities in recent years, specifically in China. Most studies evaluating the transformation effect of resource-based cities focus on economic transformation, while covering environmental efficiency as well as resource and energy use efficiency [44–46]. However, the case studies lack relevance, and the economic-centered evaluation system tends to breed the undesirable view of "GDP only" or “brown growth” [47]. The evaluation from a single perspective is also one-sided and not conducive to the long-term and sustainable development of resource-based cities [48]. Moreover, resource-based provinces and cities show a lack of research on the spatial and temporal distribution of the development. To fill this research gap, this study investigates a specific type of resource-based cities in the northeastern region. For this investigation, this paper constructs an index system for evaluating the development status of seven major resource-based prefecture-level cities in Jilin province. To this end, this research applies AHP and optimized entropy-weighted TOPSIS models based on the five development concepts of "innovation, coordination, green, openness, and sharing". Based on identifying their development shortcomings, this paper offers recommendations for the transformation and development of resource-based cities in Jilin province, considering the actual situation of the region and the transformation experiences in China and the globe, as well as providing a reference for other cities of the same type around the world.

## Methodology and data

### Research objects

According to the National Sustainable Development Plan for Resource-oriented Cities (2013–2020) and the Report on Sustainable Development of Resource-oriented Cities in Jilin province [49, 50], this paper defines 31 cities based on resources (counties and districts), whose land area, GDP, and population account for 70%, 50.6%, and 49% of Jilin province, respectively. The spatial distribution of resource-based cities in Jilin province has an apparent regularity. The eastern region includes forestry resource-based cities, the central area covers coal and metal resource-based towns, and the western area involves petroleum resource-based cities. This study collects data from seven resource-oriented prefecture-level cities in the northeastern area of Jilin province to provide a comparative analysis of the development characteristics of resource-based cities.

### Constitution of the evaluation index

Selecting indicators for the transformation of cities based on resources has certain special requirements. These indicators should reflect not only the current progress of resource-oriented cities in a comprehensive, scientific, and reasonable manner but also the process and effect of urban transformation in a relatively accurate manner. According to previous studies, constructing the index system of resource-oriented cities’ transformation should follow the principles of systematization, feasibility, quantification, comparability, and dynamism. Accordingly, Table 1 shows five indicators including population, investment, industry, ecology, and society as the primary indicators in the index system. Under these primary indicators, Table 1 represents 12 secondary sub-indicators involving employment, average wage, fixed assets, foreign direct investment (FDI), industrial organization, industrial growth, green ecology, non-green ecology, health care, education, patents, and social security.

**Table 1:** Index system for evaluation.

	Evaluation Index	Mean	Max	Min
**Panel A. population**				
**Employment**	Number of people employed in urban units (10,000)	34.27	154.01	8.73
	Registered urban unemployment (10,000)	2.45	8.82	0.64
**Average salaries**	Total wages paid in the reporting period / average number of all employees in the reporting period (10,000 yuan)	3.04	8.81	0.56
**Panel B. Investment**				
**Fixed Assets**	Investment in fixed assets (excluding farm households) (100,000,000 yuan)	774.48	5194.80	235.20
**Foreign Direct Investment (FDI)**	Real foreign investment (1,000,000 yuan)	422.17	6497.92	-424.31
**Panel C. Industry**				
**Industry Structure**	Regional GDP (100,000,000 yuan)	1139.57	7175.71	63.70
	Gross regional product per capita (10,000 yuan)	3.21	9.57	0.46
	Real GDP (100,000,000 yuan)	724.03	3812.91	62.42
	Real GDP per capita (10,000 yuan)	2.03	5.08	0.46
	Gross secondary industry/GDP (Percent)	6.68	60.38	19.58
	Persons employed in urban units at the end of the year in the secondary sector (10000)	6.97	72.57	2.57
	Gross Tertiary Sector/GDP (Percent)	45.88	60.38	19.58
	Tertiary sector year-end employment in urban units (10000)	14.19	72.57	2.57
	Persons employed in urban units in the tertiary sector at the end of the year / Total number of workers (Percent)	37.86	61.52	20.96
**Industrial Growth**	Real GDP growth rate (Percent)	17.94	55.65	-47.00
	Real GDP per capita growth (Percent)	53.44	55.19	-47.00
**Panel D. Ecology**				
**Green Ecology**	Urban green areas (Hectare)	4062.51	20750.00	465.00
**Non-Green Ecology**	Industrial wastewater discharge (104t)	4094.02	18692.00	30.00
**Panel E. Society**				
**Education**	Total number of students on campus (100,000,000)	34.48	102.92	8.00
	Number of schools at all levels	966.03	5908.00	226.00
	Ratio of teachers and students (Percent)	23.18	28.70	16.68
**Healthcare**	Number of hospital beds (10,000)	1.30	5.13	0.29
**Patents**	Number of patents	978.03	10268.00	115.00
**Social Security**	Consumer price of living index (Percent)	4.82	31.20	0.23
	Number of participants in pension insurance at all levels (10,000)	65.97	241.28	0.57

The industrial indicator system evaluates the effect of economic change in resource-oriented cities. According to the theory of industrial structure and life cycle, the economic transition of resource-oriented cities is mainly the adjustment of the leading industries. This adjustment implies that the industrial structure changes with government intervention and capital investment, and the industrial growth shows a new trend and enters a new cycle. Therefore, the measurement of the effect of economic growth in resource-oriented cities has two secondary indicators: industrial structure and industrial growth indicators.

Investment indicators evaluate the impact of external factors, mainly non-spatial factors, received by resource-oriented cities in the stages of transformation and development. Hence, investment indicators have two secondary indicators: direct investment by cities and counties and foreign investment.

The indicator system of society and population mainly evaluates the changes in social development in resource-oriented cities within the stages of transformational development. According to sustainable development theory, positive social development means that the overall society develops in an equitable and harmonious way. In other words, sustainable social development infers that the social class structure improves while urban development and people’s living standard are increasing. Therefore, demographic indicators have two secondary indicators: the employment rate and the average wage. Also, social indicators have four secondary indicators: education, healthcare, patents, and social security.

The theory of sustainable development implies that economic transformation involves a harmonious development of both the economy and the environment. This sustainable transformation strengthens the protection of the environment in development, and reduces the damage and consumption of the environment. Therefore, the evaluation of environmental development has two secondary indicators: green and non-green ecological indicators.

### Data resources

The research data were mainly obtained from the China Urban Statistical Yearbook 2000–2019, the Statistical Bulletin of National Economic and Social Development of Cities, the Chinese government’s State Council on the Issuance of the National Sustainable Development Plan for Resource-oriented Cities (2013–2020), and Development in North East People’s Republic of China: An analysis of enterprise performance 1995–2002. This research estimates the missing data using the average growth rate method.

### Research methods

This study uses the entropy-weighted TOPSIS measurement as the main analysis method. Entropy-weighted TOPSIS combines the traditional TOPSIS with entropy method, a commonly used evaluation model for assigning table weights. This approach reflects the contrast and resolution among indicators comprehensively, effectively avoiding difficulties in analysis and evaluation caused by too small data differences, and can reflect the utility of indicator information in a comprehensive and systematic way [51]. Then, this research employs the Analytic Hierarchy Process (AHP) method for the robustness test. The AHP is the research method created by American operations researcher Sadie, which divides complex problems into various levels of factors. This method analyzes each factor two by two to rank each factor at each level. Then, this technique applies methods such as judgment matrices to calculate the weights of the relative order of importance of each level element, integrates qualitative and quantitative analysis, and is a powerful tool for analytical decision-making. Moreover, the Moran index explores spatial autocorrelation to check whether a clustering effect exists among cities in Jilin province.

#### TOPSIS method

In 1981, Hwang and Yoon proposed the TOPSIS as a multi-attribute decision-making method. This method focuses on the multi-objective evaluation and decision analysis of finite solutions as a ranking method for approximating ideal solutions. This method ranks solutions according to their closeness to the positive ideal solution and distance from the negative ideal solution. The original TOPSIS method has the same indicator weight, which does not reflect the relative importance of the indicators. The combination of the entropy method, which objectively determines the indicator weight, with the TOPSIS method is an appropriate way of weighting the indicators [52]. This paper constructs the following steps to construct the entropy-TOPSIS evaluation model.

(1) *Establishing the evaluation matrix*: The traditional TOPSIS method can only evaluate the relative strengths and weaknesses of studied samples, and cannot be compared with objects outside the sample. This paper introduces the data of major cities based on resources in Jilin province between 2000 and 2019 to compare the relative level of transformation development among cities based on resources in Jilin province, as well as to compare the development gap among cities based on resources in the region in different years. Eq 1 represents the judgment matrix *X*, assuming *m* evaluation units and *n* evaluation indicators.

$$X={\left(x_{ij}\right)}_{m\times n},(i=1,2,\cdots ,m;j=1,2,\cdots ,n)$$ (1)

(2) *Initial data normalization*: The extreme value method is used to dimensionless process the initial data to obtain the judgment matrix *Q*, with the evaluation indicator *q*_*ij*_:

$$Q={\left(q_{ij}\right)}_{m\times n},(i=1,2,\cdots ,m;j=1,2,\cdots ,n)$$

(3). *Determining the indexes’ weights*: The information entropy *H*_*j*_ of the index n is collected to determine the weighting *w*_*j*_.

$$\begin{array}{c} H_{j}=-\frac{1}{\ln\;m}\sum_{i=1}^{m}P_{ij}\;\ln\;P_{ij}\\ P_{ij}=q_{ij}/\sum_{i=1}^{m}q_{ij}\\ w_{j}=\left(1-H_{j}\right)/\sum_{j=1}^{n}\left(1-H_{j}\right) \end{array}$$ (2)

(4) *Calculating optimal and inferior solutions*: Eq 3 calculates the weighted judgment matrix *R*:

$$R=w_{j}Q={\left(r_{ij}\right)}_{m\times n}$$

Where *w*_*j*_ is the weight of evaluation indicator *j*.

Eq 3 determines the optimal $r_{j}^{+}$ and inferior $r_{j}^{-}$.


$$\begin{array}{c} r_{j}^{+}=max(r_{1j},r_{2j},\cdots ,r_{mj})\\ r_{j}^{-}=min(r_{1j},r_{2j},\cdots ,r_{mj}) \end{array}$$ (3)

Eq 4 calculates Euclidean distances.


$$\begin{array}{c} {sd}_{i}^{+}=\sqrt{\sum_{j=1}^{n}{\left(r_{j}^{+}-r_{ij}\right)}^{2}}\\ {sd}_{i}^{-}=\sqrt{\sum_{j=1}^{n}{\left(r_{j}^{-}-r_{ij}\right)}^{2}} \end{array}$$ (4)

In Eq (4), a small ${sd}_{i}^{+}$ shows that the evaluation object is close to the positive ideal solution, and the urban transformation is appropriate and consistent with sustainable development. In contrast, a low value of ${sd}_{i}^{-}$ shows that the evaluation object is close to the negative ideal solution, and the urban transformation is inappropriate and inconsistent with sustainable development.

*(5) Calculating the composite index*: Eq 5 calculates the composite index.

$$D_{i}={sd}_{i}^{-}/\left({sd}_{i}^{+}+{sd}_{i}^{-}\right)$$ (5)

In Eq (5), 0≤*D*_*i*_≤1, and the larger the value, the more appropriate the urban transformation in year *i*.

#### AHP

The basic idea of the hierarchical analysis method of AHP proposed by Professor T. L. Saaty of the University of Pittsburgh is to decompose a complex problem into its constituent elements, determine the relative importance of each element through comparison, and then form a comprehensive judgment of the importance of each element. The AHP method calculates the weight of each indication of the aforementioned urban complete evaluation index system.

(1) *Constructing a judgment matrix*

Eq 6 represents the basic form of the judgment matrix.


$$C=\{\begin{array}{cccc} 1 & C_{12} & \ldots & C_{1n}\\ C_{21} & 1 & \ldots & C_{2n}\\ \vdots & \vdots & \ddots & \vdots \\ C_{m1} & C_{m2} & \ldots & 1 \end{array}\}$$ (6)

In Eq (6), *C*_*ij*_ is the relative importance of element *i* to element *j*. As specified by the matrix judgment scale, *C*_*ij*_ takes values from 1 to 9 and must satisfy: *C*_*ii*_ = 1, *C*_*ij*_−1/*C*_*ji*_, *C*_*ij*_>0.

(2) *Calculating the weights of the first-level indicators*

MATLAB software calculates the maximum characteristic roots of the judgment matrix. Eq 7 computes the consistency indicators to perform the judgment matrix’s consistency test.


$$CI=\frac{\lambda_{\max}-n}{n-1}=\frac{5.0522-5}{5-1}=0.0131$$ (7)

Where *CI* is the column normalization index.

Eq 8 shows the stochastic consistency ratio, *CR*.


$$CR=\frac{CI}{RI}=\frac{0.0131}{1.12}=0.0117<0.10$$ (8)

Where *RI* is the random consistency index.

According to the results, the allocation of the weight coefficients is extremely logical, and the hierarchical analysis is thus consistent. MATLAB computes the weight of level 1 indicators.

(3) *Calculation of weights for secondary indicators*

The judgment matrix *S* = (*u*_*ij*_)_*p*×*p*_ can be constructed, and MATLAB software can be used to calculate the maximum characteristic roots of the judgment matrix to obtain, *λ*_max_ = 2. MATLAB software can calculate the weights of the indicators.

(4). *Consistency test*

MATLAB software can calculate the maximum eigenvalue of the judgment matrix. In order to conduct the consistency test of the judgment matrix *λ*_max_ = 4.0042, Eq 9 calculates the consistency indicators.


$$CI=\frac{\lambda_{\max}-n}{n-1}=\frac{4.0042-4}{4-1}=0.0014$$ (9)

According to the average stochastic consistency indicator which is *RI* = 0.89, Eq 10 calculates the stochastic consistency ratio as follows.


$$CR=\frac{CI}{RI}=\frac{0.0014}{0.89}=0.0016<0.10$$ (10)

Therefore, the resulted hierarchical analysis is consistent, confirming that the distribution of the weight coefficients is very rational. After the hierarchical analysis method, this study obtains the relative weight values of the 25 indicators for assessing the development level of resource-oriented cities. It is only necessary to obtain the score value of all indicators for each city, then multiply it by the corresponding weight, and sum the scores to obtain the development level scores of the seven resource-oriented cities in Jilin province between 2000 and 2019.

#### Moran index

Tobler’s first law of geography is the basis of the fundamental starting point of spatial correlation analysis, which states that everything is related to everything else, but things that are close together are more closely related. In order to check the spatial correlation of the declining trend of major cities in Jilin province, this study takes the commonly-used Moran’s index as a statistical measure for analysis. Moran’s index is classified into the global Moran’s I, which reflects whether there is a vital spatial correlation in the whole region, and the Local Moran’s I, which indicates the clustering characteristics of each spatial element in the region.

(1) *Global Moran index I*.

The global Moran index, proposed by Moran in 1950, is the correlation coefficient between what can be thought of as an observation and its spatial lag [41], represented in Eq 11.

$$I=\frac{\sum_{i=1}^{n}\sum_{j=1}^{n}w_{ij}\;(x_{i}‐\bar{x})\;(x_{j}-\bar{x})}{S^{2}\sum_{i=1}^{n}\sum_{j=1}^{n}W_{ij}}$$ (11)

where *N* represents the total number of areas in the study area, *W*_*ij*_ is the spatial weight, *X*_*i*_ and *X*_*j*_ are the attributes of area *i* and area *j*, respectively, and *X* is the mean value of the attributes. Global Moran index values range from—1 to 1. The positive values of this index indicate that attributes with similar characteristics are clustered together, while its negative values imply that attributes with different characteristics are clustered together. If the values of this index are close to zero, the attributes have a random distribution without any spatial autocorrelation.

(2) *Local Moran index I*

Anselin proposed the local Moran index in 1995 to measure the degree of association between region i and its domain [53], based on Eq 12.


$$I_{i}=\frac{(X_{j}-\bar{X})\sum_{j}\left[W_{ij}\;(X_{i}-\bar{X})\right]}{\sum_{j}{\left(X_{j}-X\right)}^{2}/N}$$ (12)

A positive value of the local Moran index infers that a high value is surrounded by a high value (high-high), or a low value is surrounded by a low value (low-low). In contrast, a negative value implies that a low value is surrounded by a high value (low-high), or a high value is surrounded by a low value (high-low).

## Result

### Measurement of urban development and decline

The calculations based on the entropy-weighted method show that Baishan City reached its maximum value in 2019 for the last 20 years, as did the cities of Siping, Songyuan, and Tonghua. However, the difference between the maximum and the corresponding minimum values is very small and fluctuates around 0.01 in the last 20 years, even after reaching the maximum values. Changchun, Jilin, and Liaoyuan all reached their maximum values in 2014 and have been on a downward trend ever since. The maximum value is about twice the minimum value.

**Fig 1:**
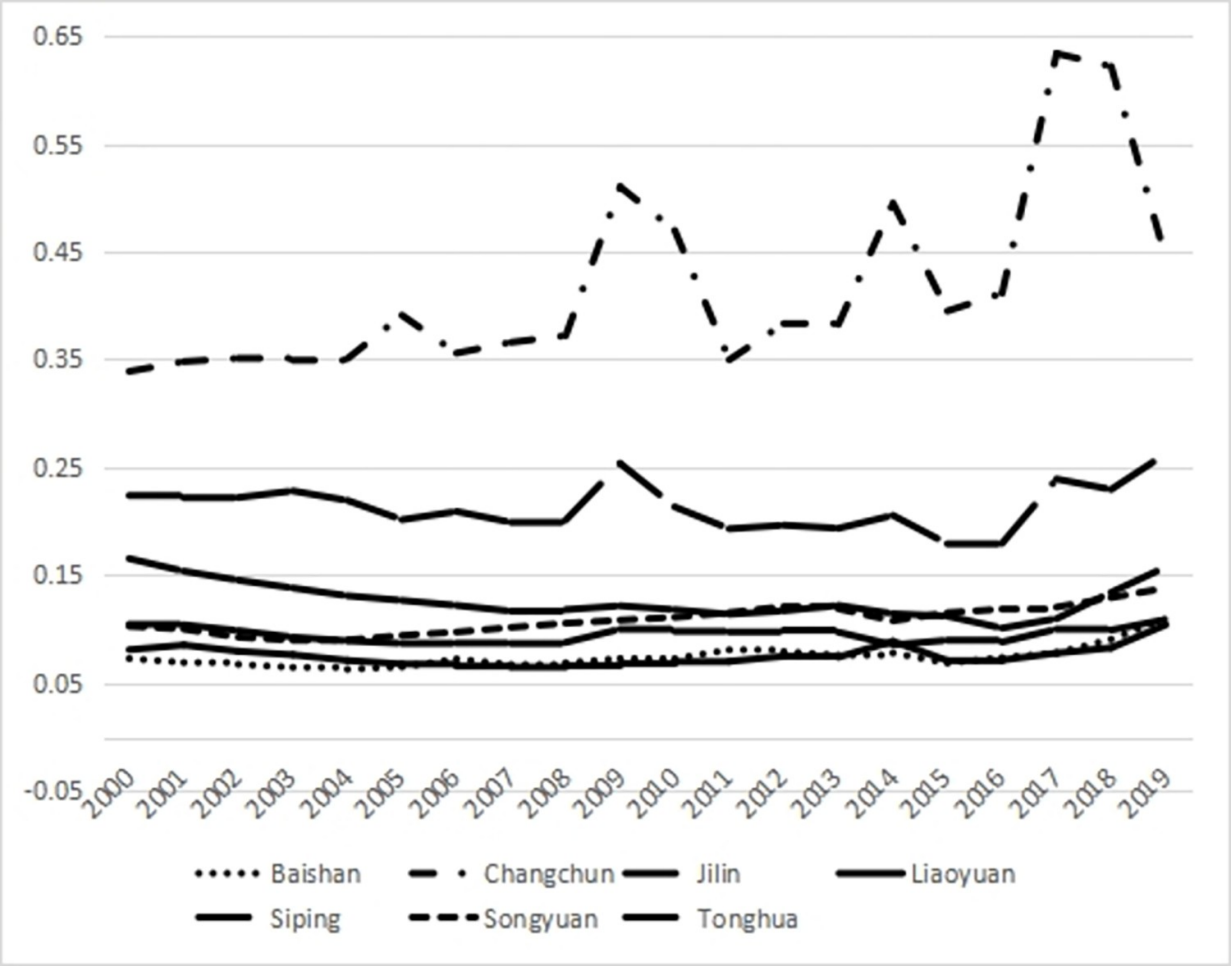
Results of the superior and inferior solution distance method shown in line chart.

**Fig 2:**
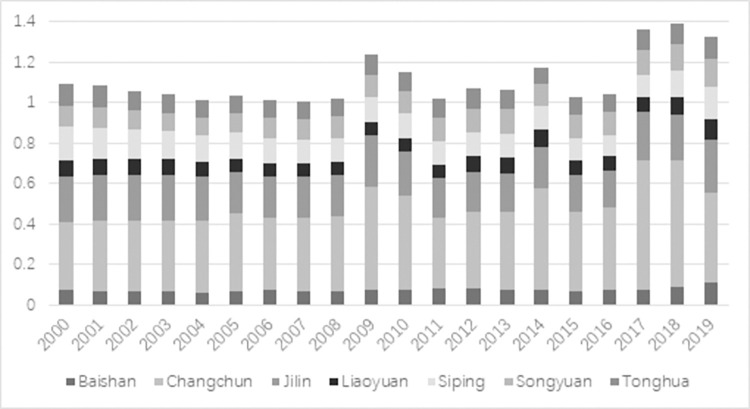
Results of the superior and inferior solution distance method shown in bar chart.

According to the entropy-weighted TOPSIS model, this research assesses and rates the closeness of the main resource-oriented cities in Jilin province to transformation. Table 2 shows the overall proximity to the transformation of cities based on resources in Jilin province from the highest to the lowest: Changchun, Jilin, Tonghua, Songyuan, Siping, Liaoyuan, and Baishan. Among them, Songyuan and Tonghua are identical and both of them capture the third place. In general, Figs 1 and 2 show that, except for Changchun, the average value of comprehensive closeness fluctuates around 0.2 from 2000 to 2019. This value is much less than 1, which implies that the level of transformation based on resources is under the expected levels, indicating much room for further transformation of the cities. The difference in proximity between the first and second-ranked cities is two times the mean value, indicating a big gap between the transformation effect of other cities in Jilin province and that of Changchun. The transformation shows a decline in all cities during 2005 and 2012, then it rises to reach a peak in 2015 to show the best performance in this year compared with the last 20 years. Since then, this trend resumes a downward trend until the end of the period. Changchun and Jilin have lower combined proximity values in 2019 than those in 2000, while Tonghua, Songyuan, Siping, and Baishan have slightly higher combined proximity values in 2019 than those in 2000. However, the overall level is still relatively low. The population and area of the cities are shown in Table 3.

**Table 2:** Results of the superior and inferior solution distance method.

	Baishan	Changchun	Jilin	Liaoyuan	Siping	Songyuan	Tonghua
**2000**	0.072687	0.338973	0.223889	0.080933	0.165362	0.102685	0.104493
**2001**	0.068993	0.348079	0.221851	0.085261	0.153738	0.100061	0.103972
**2002**	0.06732	0.351112	0.222027	0.079522	0.145393	0.092531	0.098623
**2003**	0.064485	0.349277	0.228138	0.076232	0.138200	0.089703	0.092584
**2004**	0.062731	0.350843	0.219312	0.070997	0.130926	0.089927	0.089150
**2005**	0.064963	0.390958	0.201494	0.067688	0.126652	0.094153	0.086861
**2006**	0.072370	0.355911	0.209101	0.065771	0.122025	0.097280	0.087109
**2007**	0.067137	0.36592	0.199050	0.064867	0.116667	0.101624	0.086732
**2008**	0.068768	0.372108	0.201153	0.066271	0.118069	0.105472	0.087766
**2009**	0.072779	0.509931	0.253520	0.068070	0.121657	0.108313	0.100029
**2010**	0.072671	0.469611	0.213321	0.069841	0.118045	0.111002	0.097977
**2011**	0.080765	0.349750	0.193048	0.070138	0.113834	0.115331	0.097455
**2012**	0.079227	0.383256	0.196351	0.074961	0.117015	0.120929	0.099094
**2013**	0.075843	0.382634	0.193488	0.074066	0.122011	0.118923	0.097143
**2014**	0.077865	0.495031	0.205471	0.088714	0.114336	0.107803	0.085406
**2015**	0.068459	0.394975	0.179069	0.070803	0.112049	0.115218	0.089760
**2016**	0.073673	0.411384	0.179530	0.071342	0.100987	0.118585	0.088038
**2017**	0.077975	0.633670	0.239174	0.077810	0.109712	0.120399	0.100024
**2018**	0.090495	0.621708	0.229536	0.082690	0.134064	0.129253	0.099148
**2019**	0.109511	0.444013	0.262600	0.103998	0.157580	0.138074	0.108998
**Max**	0.109511	0.633670	0.262600	0.103998	0.165362	0.138074	0.108998
**Min**	0.062731	0.338973	0.179069	0.064867	0.100987	0.089703	0.085406
SD*	0.010292	0.085903	0.021758	0.009266	0.016775	0.012950	0.006859
**Average**	0.074436	0.415957	0.213556	0.075499	0.126916	0.108863	0.095018

* SD is the standard deviation.

**Table 3:** The population and area of study area.

City	Population (2019)	Area (km^2^)
**Baishan**	1,029,800	16,795.72
**Changchun**	7,674,439	20,571.50
**Jilin**	4,465,900	20,586.42
**Liaoyuan**	1,240,000	5,125.11
**Siping**	3,428,000	14,893.10
**Songyuan**	2,025,300	22,405.70
**Tonghua**	2,324,400	15,110.00

**Fig 3:**
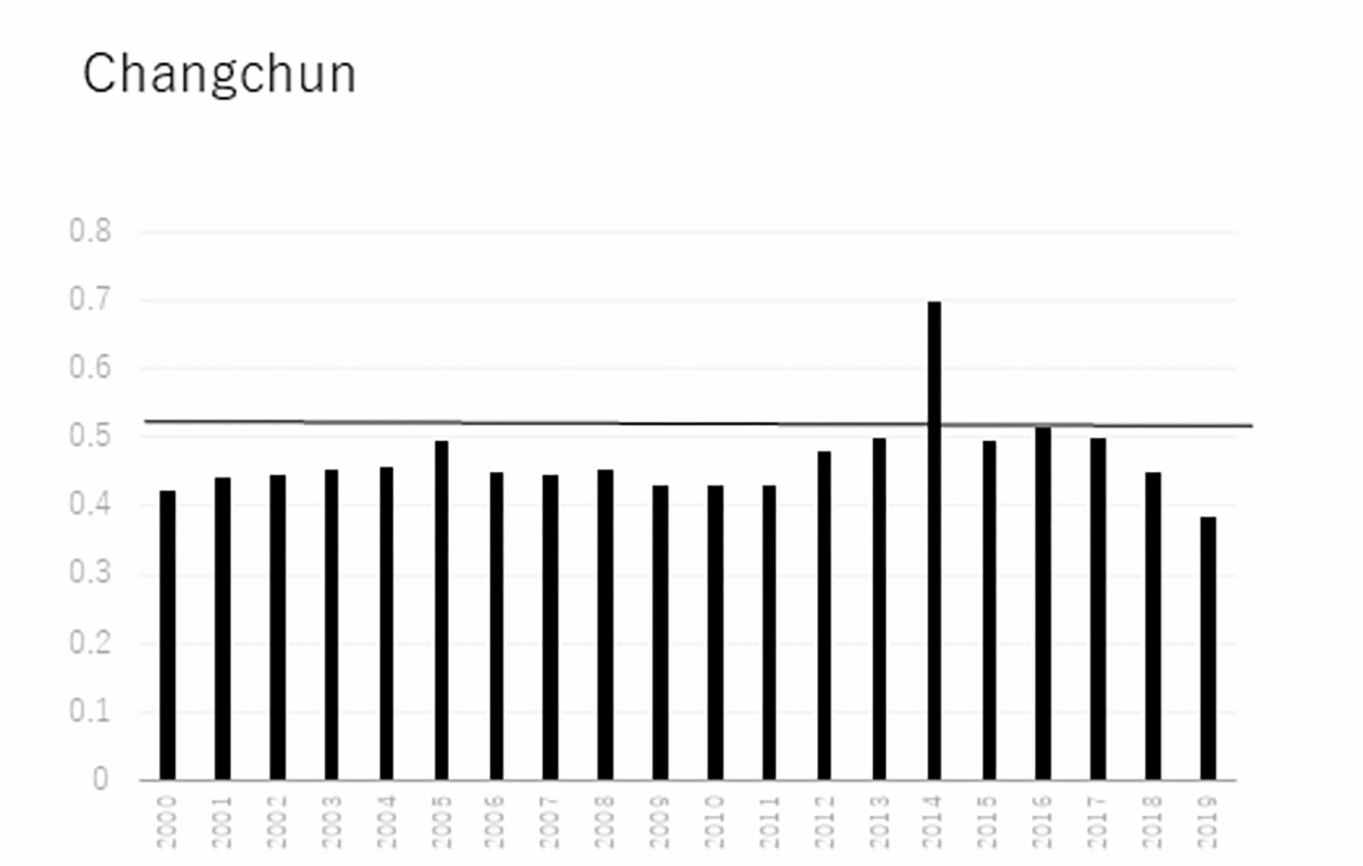
Calculation results of Changchun from 2000 to 2019.

**Fig 4:**
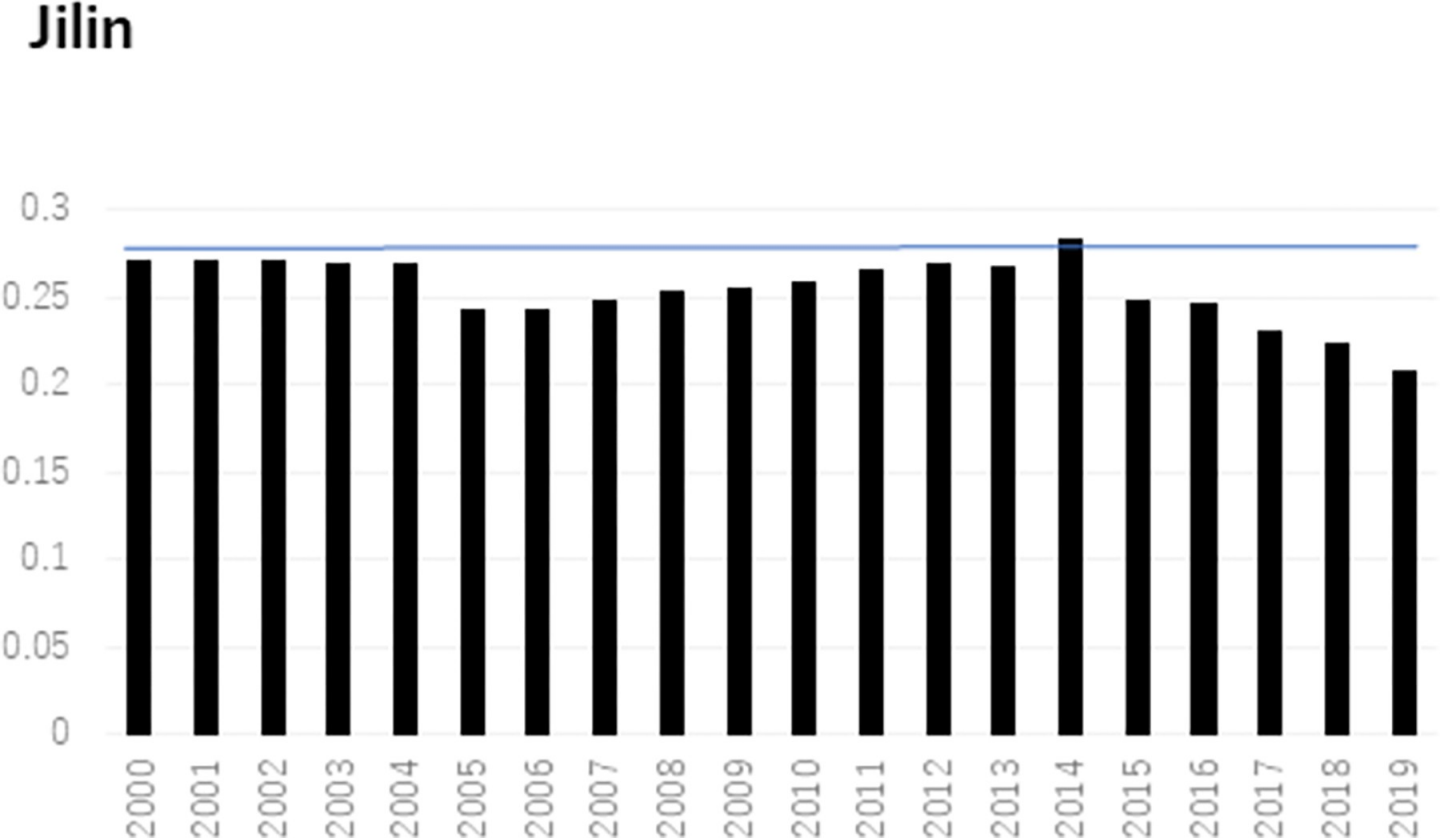
Calculation results of Jilin from 2000 to 2019.

In terms of individual city development, Fig 3 displays that the development of Changchun is low and fluctuates from 2000 to 2011. However, this value begins to rapidly develop in 2011 until reaching its maximum in 2014. Since then, the development trend reverses and shows a decreasing pattern. This trend maintains above the 20-year average within 2011 and 2018, showing its effectiveness. Then, this trend falls below the average due to the significant effect of an array of influencing factors in 2019. As the only more developed city in Jilin province, as shown in Fig 4, Jilin city also shows a similar trend to Changchun.

**Fig 5:**
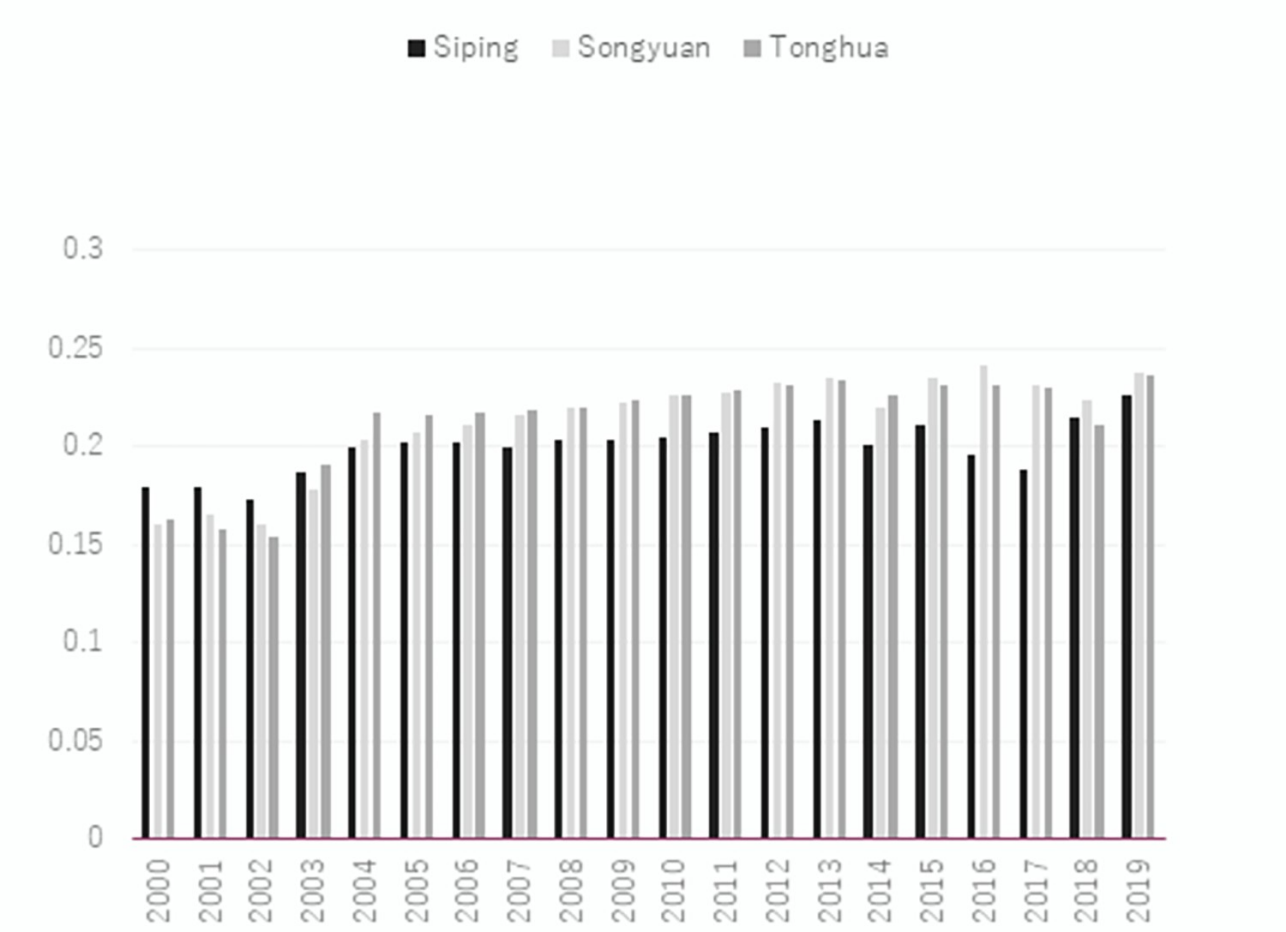
Calculation results of Siping, Songyuan, and Tonghua from 2000 to 2019.

**Fig 6:**
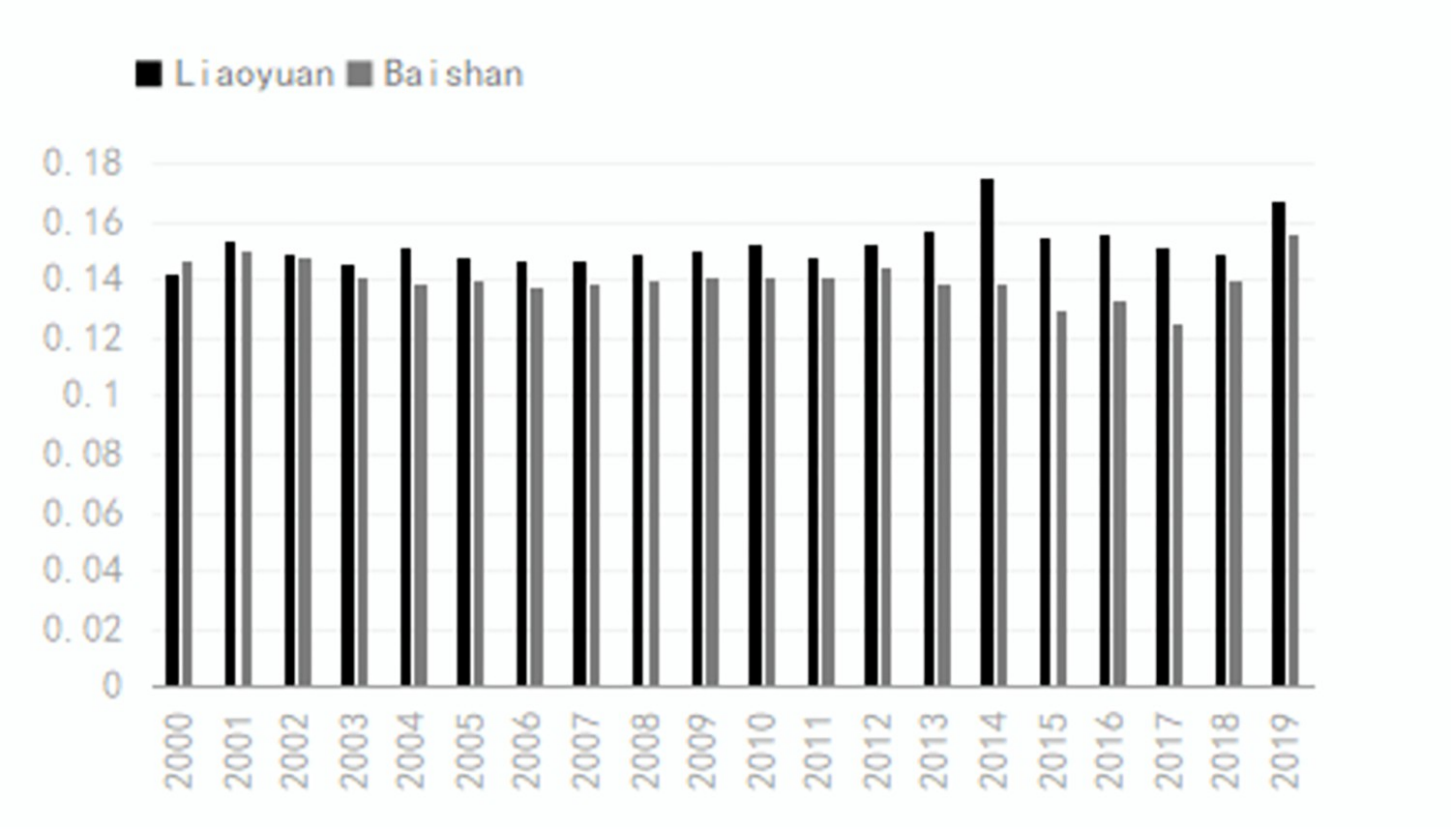
Calculation results of Liaoyuan and Baishan from 2000 to 2019.

According to Fig 5, Siping, Songyuan, and Tonghua showed a downward trend from 2000 to 2003, then an upward trend until 2013, and an upward trend from 2013 to 2019, despite a partial decline. With regard to Fig 6, Liaoyuan and Baishan show a similar trend, with some fluctuations but an overall upward trend. Despite a slightly upward trend in urban development, urban development overall shows a low level with a large gap among cities in the province. The province’s average development still shows a downward trend, despite an upward trend.

### Robustness analysis

#### Comparison of AHP and entropy-weighted TOPSIS method

The primary indicators have the following ranking: population, industry, society, investment, and ecology. Among the population indicators, the average wage has the highest weight; among the industry indicators, the weight of industrial growth is the highest; among the social indicators, the weight of health care is the highest; among the investment indicators, the weight of fixed assets is the highest; and among the ecological indicators, the weight of green ecology is the highest. Using the hierarchical analysis method to reveal the evaluation index system of urban development in Jilin, the weight of each indicator, and the size of its weight is the degree of influence of the indicator on the assessment of urban transformation in Jilin province so that it can target to enhance the ability of the indicators with greater weight, to provide a scientifically theoretical basis for the progress of cities based on resources. Table 4 shows the calculated weights of each indicator. The score of each indicator for each city in each year must be multiplied by the corresponding weight to get the score. Then, the score will be summed up to obtain the development level score for each of the seven cities from 2000 to 2019, while the results are listed in descending order according to the score. Table 4 represents the results in more details.

**Table 4:** Weighting of evaluation index using AHP method.

Subsystem				Evaluation Index	Weighting	Combined Weighting
Population	0.388	Employment	0.333	Number of people employed in urban units	0.333	0.043
				Registered urban unemployment	0.667	0.0862
		Average salaries	0.667	Total wages paid in the reporting period / average number of all employees in the reporting period	1	0.2588
Investment	0.125	Fixed Assets	0.75	Investment in fixed assets (excluding farm households)	1	0.0938
		Foreign Direct Investment (FDI)	0.25	Actual foreign investment	1	0.0313
Industry	0.205	Industry Structure	0.2	Regional GDP	0.0446	0.0018
				Gross regional product per capita	0.0726	0.0030
				Real GDP	0.0634	0.0026
				Real GDP per capita	0.1081	0.0044
				Gross secondary industry/GDP	0.0945	0.0039
				Persons employed in urban units at the end of the year in the secondary sector	0.0875	0.0036
				Gross Tertiary Sector/GDP	0.1472	0.0060
				Tertiary sector year-end employment in urban units	0.1345	0.0055
				Persons employed in urban units in the tertiary sector at the end of the year / Total number of workers	0.2476	0.0102
		Industrial Growth	0.8	Real GDP growth rate	0.25	0.041
				Real GDP per capita growth	0.75	0.123
Ecology	0.103	Green Ecology	0.667	Urban green areas	1	0.0687
		Non-Green Ecology	0.333	Industrial wastewater discharge	1	0.0343
Society	0.179	Education	0.135	Total number of students on campus	0.163	0.0039
				Number of schools at all levels	0.297	0.0072
				Ratio of teachers and students	0.54	0.013
		Healthcare	0.388	Number of hospital beds	1	0.0695
		Patents	0.071	Number of Patents	1	0.0127
		Social Security	0.046	Consumer price of living index	0.25	0.0021
				Number of participants in pension insurance at all levels	0.75	0.0062

#### Measurement results

According to the AHP results in Table 5, Baishan, Jilin, Liaoyuan, and Songyuan all reached their maximum urban development in 2012, while Siping and Tonghua reached their maximum values in 2014, and Changchun’s maximum value was in 2009. Overall, all cities in Jilin province show a downward trend in their score from 2013 onwards. The largest difference is in Siping, at around 0.1, with the rest of the cities fluctuating around 0.06 between the maximum and minimum values. The difference between the first and the second can be twice as large, which displays an extremely uneven and relatively low level of development in the cities of Jilin.

**Table 5:** Calculation results of seven cities in Jilin province from 2000 to 2019 by AHP.

	Baishan	Changchun	Jilin	Liaoyuan	Siping	Songyuan	Tonghua
**2000**	0.0741	0.4406	0.2432	0.0642	0.1061	0.0997	0.1039
**2001**	0.0728	0.4561	0.2434	0.0599	0.1083	0.1010	0.1042
**2002**	0.0708	0.4648	0.2481	0.0578	0.1071	0.0981	0.1031
**2003**	0.0696	0.4691	0.2543	0.0599	0.1075	0.1008	0.1016
**2004**	0.0596	0.4545	0.2511	0.0594	0.1081	0.1047	0.0983
**2005**	0.0759	0.4456	0.2076	0.0670	0.1118	0.1151	0.0936
**2006**	0.0722	0.4087	0.2077	0.0668	0.1094	0.1217	0.0957
**2007**	0.0724	0.3984	0.2174	0.0660	0.1111	0.1246	0.0965
**2008**	0.0753	0.4017	0.2250	0.0692	0.1150	0.1348	0.1014
**2009**	0.0802	0.4030	0.2335	0.0737	0.1169	0.1370	0.1060
**2010**	0.0778	0.3939	0.2303	0.0716	0.1152	0.1395	0.1050
**2011**	0.0820	0.3924	0.2303	0.0738	0.1196	0.1430	0.1094
**2012**	0.0857	0.3980	0.2333	0.0798	0.1240	0.1509	0.1129
**2013**	0.0813	0.4026	0.2333	0.0783	0.1245	0.1433	0.1133
**2014**	0.0796	0.4019	0.2129	0.0719	0.1195	0.1341	0.1061
**2015**	0.0749	0.3949	0.2110	0.0743	0.1167	0.1342	0.1050
**2016**	0.0778	0.3967	0.2082	0.0734	0.1099	0.1294	0.0975
**2017**	0.0715	0.3863	0.1870	0.0682	0.1027	0.1123	0.0966
**2018**	0.0678	0.3756	0.1659	0.0577	0.0902	0.1037	0.0782
**2019**	0.0526	0.3224	0.1268	0.0405	0.0769	0.0681	0.0706
**Average**	0.0737	0.4104	0.2185	0.0667	0.1100	0.1198	0.1000
**Ranking**	6	1	2	7	4	3	5

Table 6 uses the mean value of development for ranking the seven cities using TOPSIS and AHP methods. Based on Table 6, Changchun and Jilin are both in the first and second places in terms of development level in the province, Siping, Songyuan, and Tonghua are in 345th place, and Baishan and Liaoyuan are in the last two places. Both methods of measurement basically show the same results.

**Table 6:** Ranking the seven cities using TOPSIS and AHP methods.

	TOPSIS Rank	AHP Rank
**Changchun**	1	1
**Jilin**	2	2
**Baishan**	7	6
**Liaoyuan**	6	7
**Siping**	5	4
**Songyuan**	4	3
**Tonghua**	3	5

### Spatial effect model

Analysis of the global Moran index data in Table 7 shows that the Moran index is unequal to zero, indicating that there is still spatial correlation overall. The global Moran index is negative between 2000 and 2019, showing a negative geographical correlation but one that is insignificant, demonstrating that the spatial correlation of city development is minimal in Jilin province and that no regional development has occurred. The magnitude of the Moran index has moved towards positive values over time, showing that the cities of Jilin province are expected to form spatial autocorrelation in recent years.

**Table 7:** Results of the global Moran index of urban development in Jilin province and its significance test.

c	Moran’s I	Z-Score	P-Value
**2000**	-0.075	0.577	0.282
**2001**	-0.074	0.609	0.271
**2002**	-0.080	0.573	0.283
**2003**	-0.080	0.579	0.281
**2004**	-0.070	0.638	0.262
**2005**	-0.061	0.782	0.217
**2006**	-0.052	0.795	0.213
**2007**	-0.048	0.812	0.208
**2008**	-0.042	0.849	0.198
**2009**	-0.043	0.800	0.212
**2010**	-0.042	0.806	0.210
**2011**	-0.044	0.766	0.222
**2012**	-0.042	0.837	0.201
**2013**	-0.041	0.857	0.196
**2014**	-0.091	0.609	0.271
**2015**	-0.035	0.919	0.179
**2016**	-0.046	0.859	0.195
**2017**	-0.059	0.775	0.219
**2018**	-0.002	1.183	0.118
**2019**	-0.007	1.037	0.150

### Moran index scatter plot

**Fig 7:**
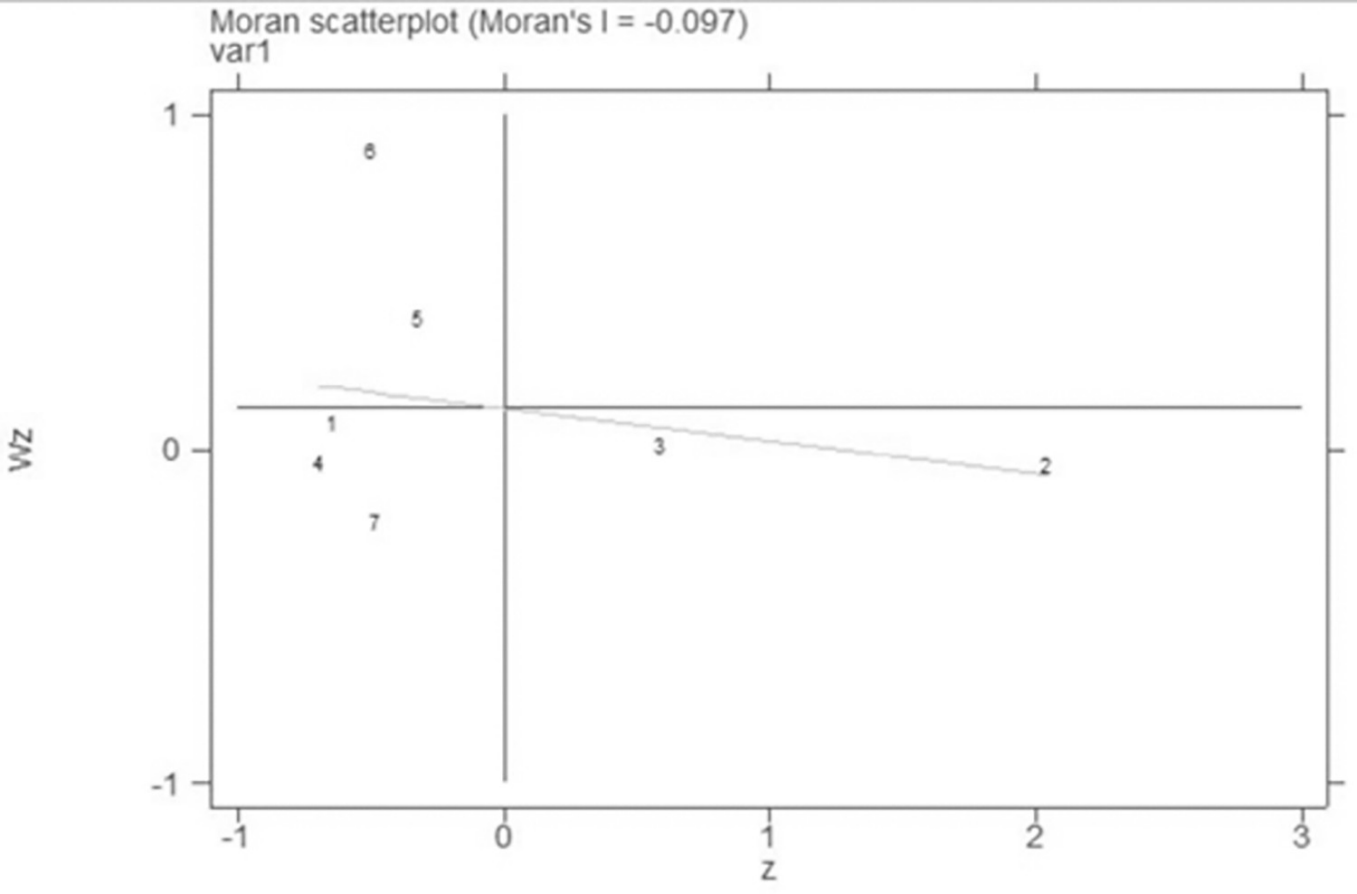
Moran index for seven cities in 2000.

**Fig 8:**
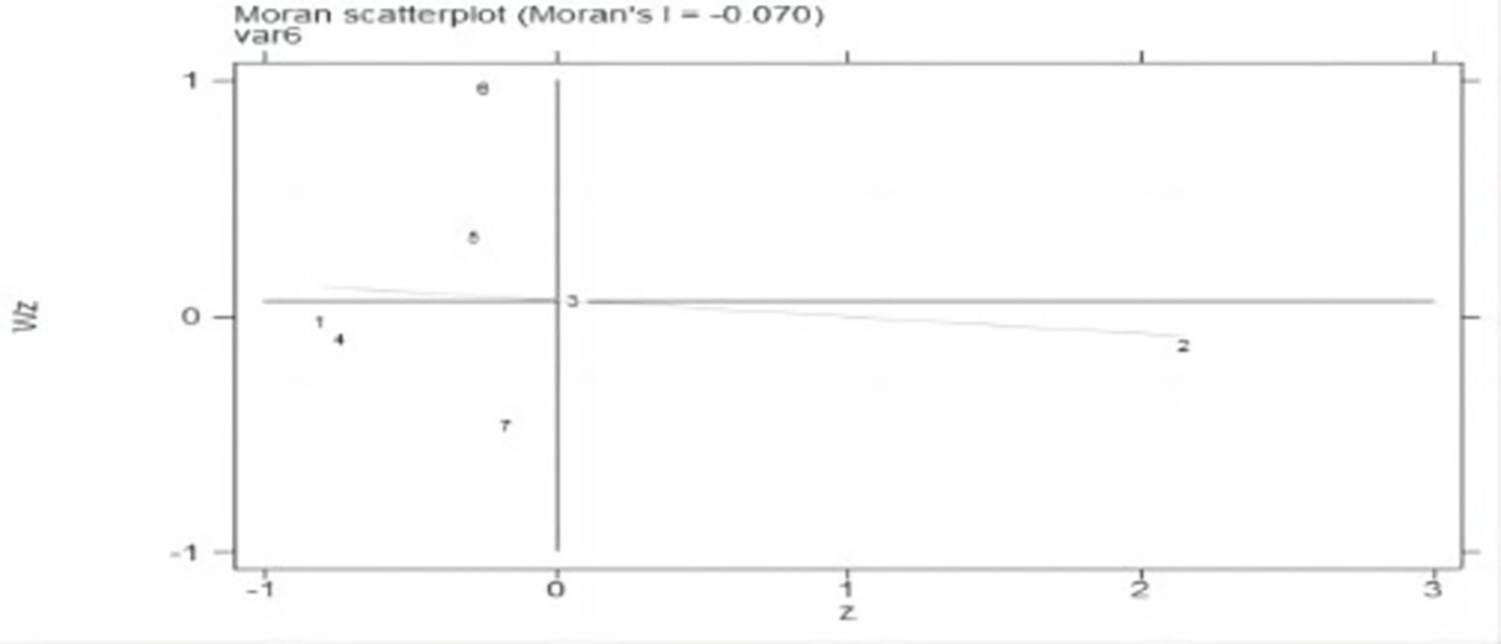
Moran index for seven cities in 2005.

**Fig 9:**
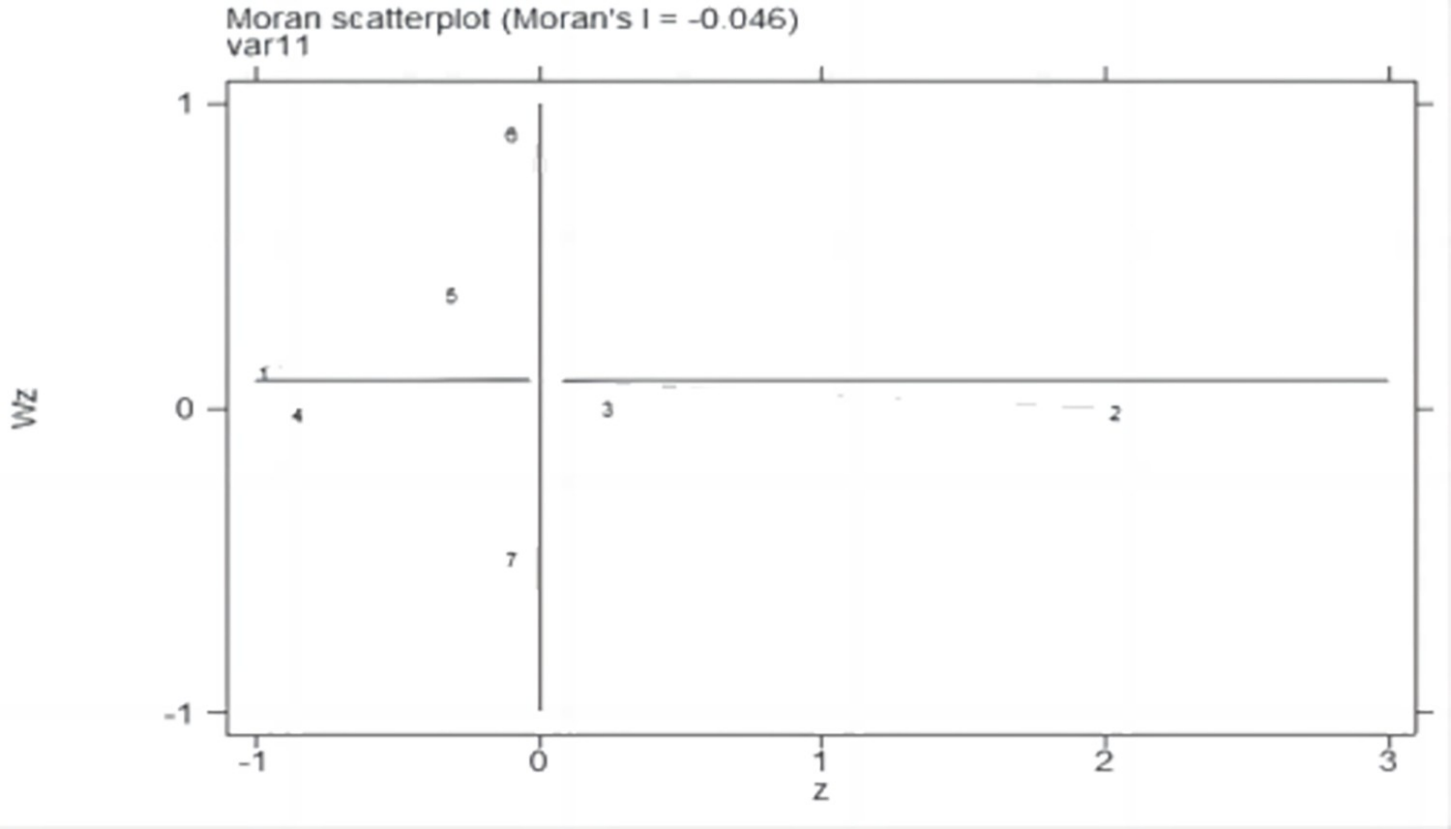
Moran index for seven cities in 2010.

**Fig 10:**
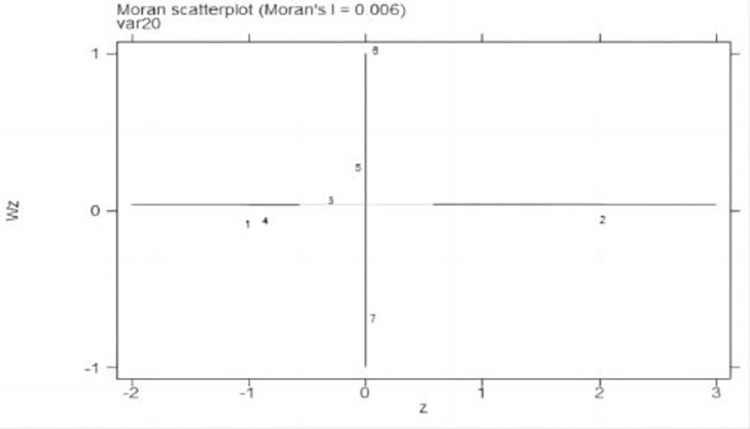
Moran index for seven cities in 2019.

This research uses the Moran index scatter diagrams and Lisa cluster diagrams to further explore the local spatial correlation characteristics of Jilin cities. Figs 7–10 show the Moran index scatter diagrams for the evaluation of urban development in Jilin province in 2000, 2005, 2010, and 2019.

According to Figs 7–10, the first quadrant is H-H type areas, while the second quadrant is L-H type areas, the third quadrant is L-L type areas, and the fourth quadrant is H-L type areas.

Based on Table 2, the cities in each quadrant of the Moran index scatter diagram do not vary greatly. L-shaped cities are Changchun and Jilin, which have a relatively high level of urban progress, while their neighboring cities have a relatively low level of urban progress. This result shows a relatively small value of the spatial correlation among the urban development of major cities in Jilin province.

## Discussion

As a resource-based province, it is difficult for Jilin to rely on its single and fixed industrial structure to sustain its long-term development. On the whole, the development shows an obvious decline trend in this province.

### Industrial perspective

The rise or development of resource-based cities like cities in Jilin is usually due to the exploitation of natural resources, and resource-dependent industries account for a large proportion of their industrial structure [54]. Due to the considerable development of science and technology, resources are losing their role as economic advantages and strengths of industries in the rapid growth of new industries and industrial transformation in developed regions. Resource-based cities are dominated by resource-exporting industries, with income distribution mostly based on contributions and intentionally repressed output efficiency and company performance. In terms of industry type, most cities are dominated by industry, agriculture, forestry, and the emerging tourism industry in recent years. In terms of capital type, state-owned enterprises are a prominent part of the economies of these cities. Resource-based cities lack foreign capital. For example, Jilin province has a limited foreign capital far less than that of the horizontal provinces according to successive yearbooks. In contrast to other regions, the lack of infusion of private capital reduces not only the economic dynamism, but also the initiative and execution of green and sustainable development in Jilin province. Considering the development of cities on the southeast coast and west of China, non-industrial private enterprises and private capital play a critical role in both economic development and environmental governance. Some private enterprises, such as Alibaba in Hangzhou, take into account the concept of green development of the city in constructing their own parks and make a direct contribution to the benign construction of the city. Also, many enterprises have contributed to the green development of their regions, providing important financial support to the city and to a certain extent of easing the contradiction between environmental protection and economic construction [55].

### Investment perspective

From an investment perspective, the lack of reserve resources is not attractive to capital. In China, some resource-oriented cities have few reserve resources available for development, and some are at the end of their development. Even if some resource-based cities have a large resource potential and are in the formative stage of development or in their heyday, there is still the problem that not all resources have been identified and need to be investigated to increase their resource reserves. Resources cannot serve as a material foundation for sustainable growth since they are ultimately limited, regardless of the reserve size. Some of the cities in Jilin province are of the combined industrial and resource type. Although these cities are able to achieve a relatively stable circular system, they need the integrity of the industrial system and strong product competitiveness to achieve overall coordinated and sustainable development. In the 20th century, however, technology and capital replaced them since the accelerated process of economic globalization has increased competition in the market with the rapid development of the scientific and technological revolution whose main feature is information technology. These changes have sharply declined the demand for resource-based and primary processed products due to internal and external factors such as the depletion of resources and the heavy historical burden. The development of resource-oriented industries and cities which are formed based on resource advantages is inevitably subject to changes in resource reserves and the supply-demand structure in the corresponding market. The development of resource-based industries, activities, and cities, formed on the basis of their resource advantages, is bound to be constrained by changes in resource reserves and the corresponding market supply and demand structures [56].

### Demographic perspective

As Popescu said, human resources have unlimited potential for growth and development [57]. From a demographic viewpoint, there are a serious population exodus and a slow growth in GDP per capita compared to the eastern coastal region. This comparative result is mainly due to the fact that in the process of economic transformation in cities based on resources, industrial restructuring has led to the bankruptcy and closure of enterprises, resulting in a large number of workers being laid off and returning to poverty. Simultaneously, the living environment of the people has been affected by many factors like coal mining sinkholes, which have delayed the improvement of the living environment of the inhabitants and to a certain extent contributed to the migration of young adults [14, 58, 59].

### Environmental perspective

From an environmental standpoint, urban pollution is increasingly becoming serious. Pollution is a significant issue brought on by the industrial design of resource-based cities. Cities that rely on natural resources and have an industrial concentration are unable to completely eliminate major air, water, and noise pollution. Today, the Jilin region is also experiencing serious haze, which is related to the emission of soot and exhaust gases from industrial production. In the process of building the city, the initial pursuit of industrial output value did not pay attention to environmental protection, and to a certain extent took the old path of "pollution first, treatment later". The city had a high density of heavy industry, flourishing small workshops with substandard emissions, which had a negative impact on ecological construction. The pollution problem, in general, has a profoundly harmful impact on the health of the city’s inhabitants, and significantly reduces the city’s attractiveness to foreigners and the cohesiveness of the existing population [14]. Also, this issue has indirectly led to a loss of the new population, with many young people choosing to leave the province. In the data report. For this reason, people find increasing aging within Jilin province, a decline in productivity, and an increase in government public expenditure on the aging population, with a consequently slowing trend of economic development.

### Social perspective

From a social perspective, people have a growing demand for infrastructure such as healthcare. As the problem of aging becomes more serious, the lack of medical resources in the province will exacerbate social conflicts and reduce the attractiveness of cities, playing a negative role in the transition and progress of new resource-oriented cities.

### Urban decline similarity

The situation of urban decline in Jilin Province is similar to other countries. Among them, the decline of small towns with mining resources is the most serious. These towns’ economic development relies heavily on mining resources. However, in recent years, the available mining resources in these towns have faced depletion, and alternative industries have not formed. Due to the small scale of the primary industry and the insufficient secondary and tertiary industries, these towns have poor employment absorption capabilities, leading to population loss and economic decline.

The scale of the decline in small towns in Jilin Province is not very large, which is consistent with the characteristics of urban decline in developed countries. Small towns in the surrounding areas of Jilin Province have been affected by economic and social decline, while the economic transformation effects of other cities and Changchun City vary greatly. Similar situations also occur in some peripheral small towns in Germany, France, and Japan. These towns often face problems such as population reduction, lack of connection with the national urban network, outdated and homogeneous residential areas, leading to a continuous decrease in "bedroom towns" in the surrounding areas, as people prefer to live in the core urban areas.

The public facilities in Jilin Province’s declining small towns are gradually aging, similar to the situation of declining towns in Europe. The population loss in small towns leads to underdeveloped local infrastructure, declining commercial services, and a large number of vacant houses. Some schools are also forced to close because they do not have enough students or teachers. Small towns have fallen into a vicious cycle in terms of tax base and income, requiring more public spending to maintain infrastructure and building environments, while the funds available for investment and maintaining social public services are decreasing.

## Conclusion

This paper studies the development of major cities based on resources in Jilin province based on the concept of sustainable development. For this aim, this study constructs and analyses an indicator system for city development using the entropy-weighted TOPSIS method, hierarchical analysis, and spatial effect models. The results show that the current transformation effect of each city based on resources in Jilin province is somewhat different from the expected one due to the low average value of the comprehensive proximity of each city. In addition, the transformation effect of other cities in the province differs significantly from that of Changchun. By dimension, the impact of change in cities dependent on natural resources reveals some erratic behavior in the economic benefit dimension. In the social development and ecological environment dimensions, the transformation of cities based on resources has achieved the following results. In the science and technology innovation and resource endowment dimensions, the change in cities based on resources has developed relatively slowly. The main resource-based cities in Jilin province have a tendency to step into decline. Also, urban development is more fragmented and cannot form a scale effect of mutual support due to the lack of agglomeration effect and the scale of gathering and development. Analyzing the statistics of the major cities based on resources in Jilin province from 2000–2019 helps governments at all levels to identify and analyze the reasons for the urban decline in resource-oriented cities in the northeast areas and put forward relevant policy recommendations. This analysis also provides theoretical and practical support for the comprehensive transformation of homogeneous resource-oriented cities across the countries or the world.

The findings of this research provide the following recommendations: modernizing the industrial setup and encouraging the green economic growth. The resource-oriented cities should expand the field of resource development and extend the resource industry chain to approach sustainable development. Changchun, for example, can expand its leading industrial chain in the direction of high precision according to its own advantages, so as to accelerate the sustainable transformation of the city.

Furthermore, the government should boost the city’s innovation ability to support the transition to a more eco-friendly future. The ability to innovate in science and technology is a major driving force for the sustainable development of local economies. Local governments need to increase the proportion of budgets on science and technology, attach importance to the training of innovative talents, and actively introduce innovative enterprises in science and technology.

Moreover, policymakers should improve the social security system to maintain stable social development. The eco-friendly transformation of cities based on resources will inevitably have an impact on some enterprises and lead to a surge in the number of jobs. The local government should provide employment opportunities and job skills training for these people as far as possible to reduce employment pressure and maintain social harmony and stable development.

Finally, the decision-makers should strengthen the environmental management and build an ecologically civilized city. The local government should raise the emission standards of the corresponding enterprises and strictly control the most polluting enterprises and projects. Also, the local government should raise awareness of ecological and environmental protection, encourage research and development of environment-protection technologies, and increase the proportion of the budget on energy conservation and environmental protection.

There are also some limitations of this paper. Firstly, the study only focuses on resource-based cities in Jilin province, so the findings may not be generalizable to all regions or countries. Second, this paper does not address the potential environmental impacts of the transformation of resource-based cities, which could be an important consideration for sustainable development. Future research can take resource-oriented cities across the country as research objects for comprehensive analysis and comparison, applied to broader urban reforms, and provide a detailed analysis of the policy implications of the findings, which could broaden its usefulness for policymakers.
